# Efficacy of Second Generation Direct-Acting Antiviral Agents for Treatment Naïve Hepatitis C Genotype 1: A Systematic Review and Network Meta-Analysis

**DOI:** 10.1371/journal.pone.0145953

**Published:** 2015-12-31

**Authors:** Thanthima Suwanthawornkul, Thunyarat Anothaisintawee, Abhasnee Sobhonslidsuk, Ammarin Thakkinstian, Yot Teerawattananon

**Affiliations:** 1 Health Intervention and Technology Assessment Program, Ministry of Public Health, Nonthaburi, Thailand; 2 Department of Family Medicine, Faculty of Medicine, Ramathibodi Hospital, Mahidol University, Bangkok, Thailand; 3 Department of Medicine, Division of Gastroenterology and Hepatology, Faculty of Medicine, Ramathibodi Hospital, Mahidol University, Bangkok, Thailand; 4 Section for Clinical Epidemiology and Biostatistics, Faculty of Medicine, Ramathibodi Hospital, Mahidol University, Bangkok, Thailand; University of North Carolina School of Medicine, UNITED STATES

## Abstract

**Background:**

The treatment of hepatitis C (HCV) infections has significantly changed in the past few years due to the introduction of direct-acting antiviral agents (DAAs). DAAs could improve the sustained virological response compared to pegylated interferon with ribavirin (PR). However, there has been no evidence from randomized controlled trials (RCTs) that directly compare the efficacy among the different regimens of DAAs.

**Aim:**

Therefore, we performed a systematic review and network meta-analysis aiming to compare the treatment efficacy between different DAA regimens for treatment naïve HCV *genotype 1*.

**Methods:**

Medline and Scopus were searched up to 25^th^ May 2015. RCTs investigating the efficacy of second generation DAA regimens for treatment naïve HCV *genotype 1* were eligible for the review. Due to the lower efficacy and more side effects of first generation DAAs, this review included only second generation DAAs approved by the US or EU Food and Drug Administration, that comprised of simeprevir (SMV), sofosbuvir (SOF), daclatasvir (DCV), ledipasvir (LDV), and paritaprevir/ritonavir/ombitasvir plus dasabuvir (PrOD). Primary outcomes were sustained virological response at weeks 12 (SVR12) and 24 (SVR24) after the end of treatment and adverse drug events (i.e. serious adverse events, anemia, and fatigue). Efficacy of all treatment regimens were compared by applying a multivariate random effect meta-analysis. Incidence rates of SVR12 and SVR24, and adverse drug events of each treatment regimen were pooled using ‘pmeta’ command in STATA program.

**Results:**

Overall, 869 studies were reviewed and 16 studies were eligible for this study. Compared with the PR regimen, SOF plus PR, SMV plus PR, and DVC plus PR regimens yielded significantly higher probability of having SVR24 with pooled risk ratios (RR) of 1.98 (95% CI 1.24, 3.14), 1.46 (95% CI: 1.22, 1.75), and 1.68 (95% CI: 1.14, 2.46), respectively. Pooled incidence rates of SVR12 and SVR24 in all treatment regimens without PR, i.e. SOF plus LDV with/without ribavirin, SOF plus SMV with/without ribavirin, SOF plus DCV with/without ribavirin, and PrOD with/without ribavirin, (pooled incidence of SVR12 ranging from 93% to 100%, and pooled incidence of SVR24 ranging from 89% to 96%) were much higher than the pooled incidence rates of SVR12 (51%) and SVR24 (48%) in PR alone. In comparing SOF plus LDV with ribavirin and SOF plus LDV without ribavirin, the chance of having SVR12 was not significantly different between these two regimens, with the pooled RR of 0.99 (95% CI: 0.97, 1.01). Regarding adverse drug events, risk of serious adverse drug events, anemia and fatigue were relatively higher in treatment regimens with PR than the treatment regimens without PR. The main limitation of our study is that a subgroup analysis according to dosages and duration of treatment could not be performed. Therefore, the dose and duration of recommended treatment have been suggested in range and not in definite value.

**Conclusions:**

Both DAA plus PR and dual DAA regimens should be included in the first line drug for treatment naïve HCV *genotype 1* because of the significant clinical benefits over PR alone. However, due to high drug costs, an economic evaluation should be conducted in order to assess the value of the investment when making coverage decisions.

## Introduction

Viral hepatitis has long been an under-recognized disease despite statistics from the World Health Organization showing that there are 1.4 million deaths from hepatitis compared to 1.6 million for HIV and 1.3 million for tuberculosis [1]. The health and economic burden of Hepatitis B virus infection was recently recognized by global public health players and a significant step forward was made via the introduction of the Hepatitis B vaccine in national immunization programs in countries at all levels of economic development [2]. However, there is no such vaccine for Hepatitis C, and thus its prevention and control rely heavily on behavioral interventions, detection and treatment. This resulted in a recent global agreement on a World Health Assembly resolution on hepatitis in May 2014 [3] that urged countries to improve access for the prevention and treatment of Hepatitis B and C.

The treatment of hepatitis C (HCV) infections has significantly changed in the past few years with the introduction of direct-acting antiviral agents (DAAs). These treatments are combined with pegylated-interferon with ribavirin (PR) and could improve the sustained virological response (SVR) compared to PR alone [4–7], particularly for those with *genotype 1*—the most prevalent type of HCV worldwide and one which does not respond well to PR [8]. Nevertheless, there has been no evidence from randomized controlled trials (RCTs) that directly compare the different regimens of DAAs and PR. Therefore, this systematic review and meta-analysis aims to address this gap by comparing the clinical efficacy and adverse drug events among the regimens of DAA plus PR, dual DAA combinations with or without ribavirin, and PR alone for the treatment naïve HCV *genotype 1*. DAAs considered in this study were only second generation DAAs because first generation DAAs (i.e. boceprevir and telaprevir) had more side effects and lower efficacy, compared to second generation DAAs.

The results of this study will be useful in informing clinical practice guidelines for HCV *genotype 1* treatment, future economic evaluations for reimbursement decisions, and new clinical studies on Hepatitis C treatment.

## Methods

### Literature search

The Medline and Scopus databases were searched since their inception up to 25^th^ May 2015 for identifying relevant studies. Reference lists of eligible studies and previous systematic reviews were also explored. Search terms and search strategies for both databases are described in Appendix A and B in S1 and S2 Appendices, respectively.

### Study selection

Two reviewers (T.A. and T.S.) independently reviewed titles and abstracts for selecting the studies. Full articles were ascertained if the decision could not be made based on titles and abstracts. Disagreement between the two reviewers was decided by consensus with a third party (A.S.).

### Inclusion criteria

RCTs published in English were included in the review if they met all of the following criteria: (1) studied patients were treatment naïve HCV *genotype 1*; (2) compared the efficacy of any pairs of the following regimens: DAA plus PR, dual DAA combinations with and without ribavirin, or PR alone. Due to lower efficacy and more side effects of first generation DAAs, this review included only second generation DAAs approved by the US or EU Food and Drug Administration at the time of writing, comprising simeprevir (SMV), sofosbuvir (SOF), daclatasvir (DCV), ledipasvir (LDV), and paritaprevir/ritonavir/ombitasvir plus dasabuvir (PrOD); (3) studies measured the outcomes as SVR at weeks 12 or 24 after the end of treatment; and (4) reported the number of patients having or not having SVR in each treatment regimen.

Studies were excluded if they only compared the efficacy between different dosages of the same treatment regimens.

### Data extraction

The baseline characteristics of the included studies (i.e. author’s names, year of publication, mean age, body mass index (BMI), and baseline HCV RNA of the study’s participants, percentages of sex, and cirrhosis) and a cross-tabulate number of patients between treatment and outcomes were independently extracted by two authors (T.A., T.S.) using structural data record forms. The corresponding authors of the included studies were contacted if there was missing or inadequate information.

### Treatment regimens of interest

Treatment regimens of interest were divided into DAA plus PR regimens (i.e. SMV plus PR, DCV plus PR, and SOF plus PR), dual DAA combinations with and without ribavirin regimens (i.e. SOF plus LDV, SOF plus SMV, SOF plus DCV, SOF plus LDV and ribavirin, SOF plus SMV and ribavirin, and SOF plus DCV and ribavirin, PrOD and PrOD plusribavirin), and PR alone.

### Outcomes

The outcome of interest were SVRs at weeks 12 (SVR12) and 24 (SVR24)—defined as HCV RNA levels lower than the detectable level specified in the eligible studies at weeks 12 and 24 after the end of treatment. Safety outcomes included anemia, fatigue, and serious adverse drug events, defined as death or serious conditions which required hospital admission.

### Risk of bias assessment

The validity of each study was assessed using the Cochrane Collaboration’s tool for assessing risk of bias in randomized controlled trials [9]. Seven domains were evaluated: sequence generation; allocation concealment; blinding of participants and personnel; blinding of outcome assessments; incomplete outcome data; selective outcome reporting; and other bias. Two reviewers (TA and TS) independently performed the risk of bias assessment. Disagreement was resolved by consensus with a third party (YT).

### Statistical analysis

A direct meta-analysis was performed for studies that had similar treatment comparisons if a total number of the studies was not less than 3 studies. Risk ratios (RR) of SVR12 and SVR24 were estimated for each study and then were pooled using the inverse variance method if there was no heterogeneity between studies; otherwise, a random effect model was applied. Heterogeneity between studies was estimated using the Q test and I^2^ statistic and was considered present if the degree of heterogeneity (I^2^) was higher than 25%. Sources of heterogeneity were explored by fitting co-variables (i.e. mean age, BMI, baseline HCV RNA, and percent cirrhosis) one by one in a meta-regression.

For indirect comparisons, treatment effects of all treatment regimens were estimated by applying a two-stage network meta-analysis as follows: first, summary data were expanded into individual patient data using ‘expand’ command in STATA. Poisson regression was applied to estimate log (RR) and variance-covariance of each treatment pairwise using mvmeta_make command. Pooled RRs and their 95% confidence interval (CI) were then estimated using a multivariate random effect meta-analysis, in which within subject-study correlation was accounted for using Riley’ method. Treatment ranking was evaluated by ranking the linear predictor of each study. For predicting the treatment effect in the future, predictive interval was estimated by taking into account for uncertainty of the whole network using the ‘intervalplot’ command. The inconsistency assumption (disagreement between direct and indirect estimations) was then tested by measuring the inconsistency factor (difference between lnRRs estimated from direct meta-analysis and indirect meta-analysis).

Incidence of adverse drug events and SVR12 and SVR24 for each treatment regimen was pooled using ‘pmeta’ command. Publication bias was assessed using Egger test and Funnel plot.

All analyses were performed using STATA version 14.0. A two-sided P value less than 0.05 was considered statistically significant for all analyses except for heterogeneity test, in which a one-sided P value less than 0.1 was applied instead.

This study was complied with the prospective protocol (see data in S1 Protocol) and Preferred Reporting Items for Systematic Reviews and Meta-Analyses (PRISMA) checklist (see data in S1 Checklist).

## Results

**Fig 1:**
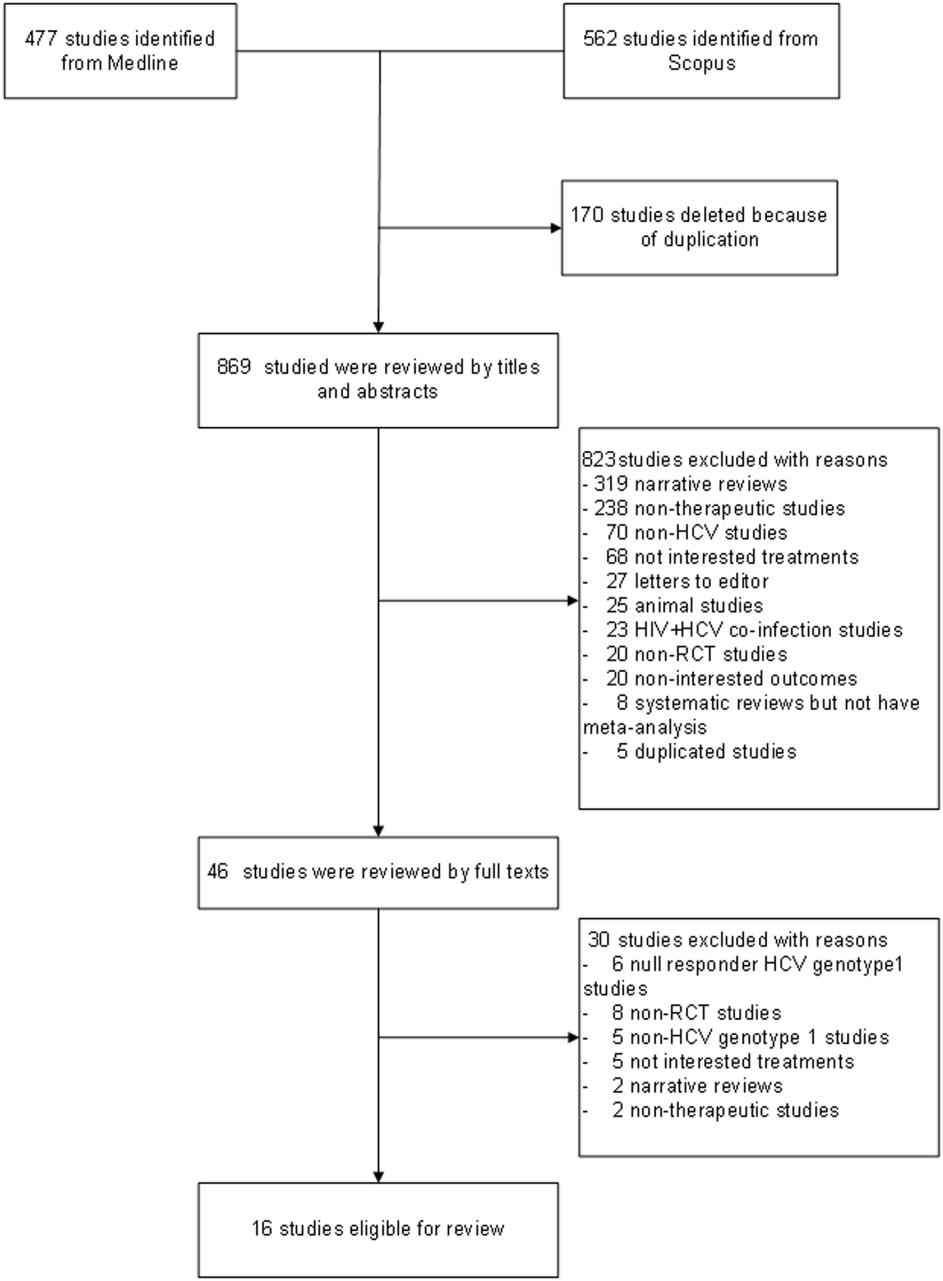
Flow chart of study selection.

We identified 477 studies from Medline and 562 studies from Scopus. After excluding duplications, 869 studies were reviewed for titles and abstracts, resulting in the final inclusion of 16 studies [4, 10–24] in the review (see Fig 1). The characteristics of the eligible studies are presented in Table 1. Among the 16 studies, 9 studies [4, 10–16, 22] assessed the efficacy of DAA plus PR versus PR alone, while 8 studies [17–21, 23, 24] assessed the efficacy of dual DAA combinations with ribavirin versus the similar regimen without ribavirin. The mean age of participants was around 40 to 50 years old and their BMI values ranged from 20 to 30 kg/m^2^. *Genotype 1a* was common in most studies except for four studies [10–12, 14] where *genotype 1b* was the most common instead. Twelve studies included only non-cirrhotic patients, while 4 studies [14, 16, 18, 23] included both cirrhotic and non-cirrhotic patients, of which the percentages of cirrhotic patients ranged from 7 to 16%. SVR was assessed at weeks 12 and 24 after the end of treatment for 15 studies [4, 11–24] and 11 studies [4, 10–16, 21, 22, 24], respectively.

**Table 1:** Characteristics of included studies.

Author	Year	Setting	Intervention	Dose (mg/day)	Duration (week)	Comparator	Dose (mg/day)	Duration (week)	Mean Age (year)	Mean BMI, (kg/m^2^)	Mean HCV RNA (log_10_ IU/mL)	Male (%)	HCV1 (%)	Cirrhosis (%)	CC/CT/TT (%)
Hayashi [10]	2013	Japan	SMV+PR^b^	50–100	12-24/ 24–48	PR^b^	-	48	50.33	-	6.17	46.8	0	0	
Fried [11]	2013	North America, Europe, Asia-Pacific	SMV+PR^b^	75–150	12-24/48	PR^b^	-	48	44.86	26.62	6.50	55.2	45.2	0	30/58/12
Hayashi [12]	2014	Japan	SMV+PR^b^	100	12/24-48	PR^b^	-	48	51.33	23.59	6.01	34.3	1.6	0	66/34^c^
Jacobso [13]	2014	Australia, North America Europe, Mexico, New Zealand	SMV+PR^b^	150	12/24-48	PR^b^	-	48	47	29.47		56.3	56.3	0	29/57/14
Manns [14]	2014	North & South America, Europe	SMV+PR^b^	150	12/24-48	PR^b^	-	48	47.35	30.59		55	41	8.4	30/54/16
Pol [15]	2012	U.S., France	DCV+PR^b^	30–60	48/48	PR^b^	-	48	51.25	-	6.50	66.8	66.8	0	35/51/14
Hézode [16]	2014	U.S., Australia, North America, Europe	DCV+PR^b^	20–60	12-24/ 24–48	PR^b^	-	48	47.55	-	6.48	67.1	75.5	7.4	30/52/12
Rodriguez-Torres [22]	2013	U.S., Puerto Rico	SOF+PR^b^	100–400	4/48	PR^b^	-	48	45	28.17	6.47	68.5	80.9	0	27/63^c^
Lawitz [4]	2013	U.S.	SOF+PR^b^	200–400	12-24/ 24–48	PR^b^	-	48	49.61	27.11	6.46	60.5	75.8	0	41/45/14
Kowdley [17]	2014	U.S.	SOF+LDV+ RBV^a^	400/90	8	SOF+LDV	400/90	8–12	52.33	28	6.43	57.7	80	0	27/57/16
Afdhal [18]	2014	U.S., Europe	SOF+LDV+ RBV^a^	400/90	12–24	SOF+LDV	400/90	12–24	50.76	29.07	6.35	59.3	67	15.7	30/52/18
Lawitz [19]	2014	U.S.	SOF+LDV+ RBV^a^	400/90	8	SOF+LDV	400/90	8–12	48.07	28.9	6.07	61.7	88	0	20/62/18
Mizokam i[23]	2015	Japan	SOF+LDV+ RBV^a^	400/90	12	SOF+LDV	400/90	12	-	-	-	40.5	-	15	-
Lawitz [20]	2014	U.S.	SOF+SMV+ RBV^a^	400/150	12–24	SOF+SMV	400/150	12–24	51.6	28.32	6.46	66.6	78.5	0	79/21^c^
Sulkowski [21]	2014	U.S.	SOF+DCV+RBV^a^	400/60	12–24	SOF+DCV	400/60	12–24	54.56	-	6.40	50.8	78.3	0	32/68^c^
Kowdley [24]	2014	U.S.	PrOD+RBV^a^	150/100/25/400	12	PrOD	150/100/25/500	12	49.21	-	6.45	58	67.3	-	-

DCV, daclatasvir; LDV, ledipasvir; PR, pegylated interferon-ribavirin; SMV, simeprevir; SOF, sofosbuvir; PrOD, paritaprevir/ritonavir/ombitasvir plus dasabuvir

^a^the dose of ribavirin was weight-base (1,000 mg/day in patients with body weight <75 kg and 1,200 mg/day in patients with a body weight ≥75 kg)

^b^the dose of pegylated interferon was 180 g/week and body weight adjusted dose for ribavirin

^C^CC/CT+TT

### Risk of bias assessment

Results of the risk of bias assessment are presented in S1 Table. All studies reported low risk of bias in the domains of blinding participants and personnel, blinding of outcome assessment, selective outcome reporting, and other bias. For the domains of random sequence generation and allocation concealment, around 25% of the studies (4/16) had unclear risk of bias and the others had low risk of bias. Two out of the 16 studies (13%) had a high risk of bias in the domain of incomplete outcome data, while the others had low risk of bias.

### DAA plus PR versus PR alone

#### Direct meta-analysis

Among all DAA plus PR regimens (i.e. SMV plus PR, DCV plus PR, and SOF plus PR), the comparison of SMV plus PR versus PR alone had sufficient data for performing direct meta-analysis of SVR12 (4 studies [11–14], n = 1,354) and SVR24 (5 studies [10–14], n = 1,252). Pooled RRs were 1.46 (95% CI: 1.28, 1.67) for SVR12 and 1.46 (95% CI: 1.26, 1.69) for SVR24, suggesting that patients receiving the SMV plus PR regimen were 46% more likely to have SVR12 and SVR24 than patients receiving PR alone (see Table 2 and S1 Fig).

**Table 2:** Pooled risk ratio of sustained virological response at weeks 12 and 24 after the end of treatment between simeprevir plus pegylated interferon-ribavirin and pegylated interferon-ribavirin.

Author	Year	SMV plus PR		PR		RR (95% CI)
		Response	Non-response	Response	Non-response	
**Sustained virological response at 12 weeks**						
Fried [11]	2013	252	57	51	26	1.23 (1.04, 1.46)
Hayashi [12]	2014	109	14	37	23	1.44 (1.17, 1.77)
Jacobson [13]	2014	210	54	65	65	1.59 (1.33, 1.91)
Manns [14]	2014	209	48	67	67	1.63 (1.36, 1.95)
**Pooled RR**						**1.46 (1.28, 1.67)**
**Sustained virological response at 24 weeks**						
Fried [11]	2013	250	59	50	27	1.24 (1.05, 1.48)
Hayashi [12]	2014	109	14	34	26	1.56 (1.24, 1.97)
Hayashi [10]	2013	63	16	6	7	1.73 (0.95, 3.14)
Jacobson [13]	2014	205	42	18	12	1.38 (1.03, 1.86)
Manns [14]	2014	206	47	28	33	1.77 (1.34, 2.34)
**Pooled RR**						**1.46 (1.26, 1.69)**

CI, confidence interval; PR, pegylated interferon-ribavirin; RR, risk ratio; SMV, simeprevir.

However, there was moderate heterogeneity as shown from the I^2^ values for both SVR12 (I^2^ = 51.7%) and SVR24 (I^2^ = 30.4%). Thus, sources of heterogeneity (i.e. age, BMI, baseline HCV RNA level) were explored but none of them could decrease the degree of heterogeneity for SVR12; however, this did not hold for SVR24, as the mean age was able to decrease the degree of heterogeneity (I^2^ decreased from 30.4 to 0%). A subgroup analysis was then performed according to age group, the pooled RRs of SVR24 were 1.42 (95% CI: 1.15, 1.76; I^2^ = 55.1%) and 1.58 (95% CI: 1.28, 1.96; I^2^ = 0.0%) for age < 50, and age ≥ 50 years, respectively (see S1 Fig). This suggested that although the treatment effect of SMV plus PR between the two age groups were not much different, its effect was more homogenous in the older than the younger age groups.

#### Indirect meta-analysis

**Fig 2:**
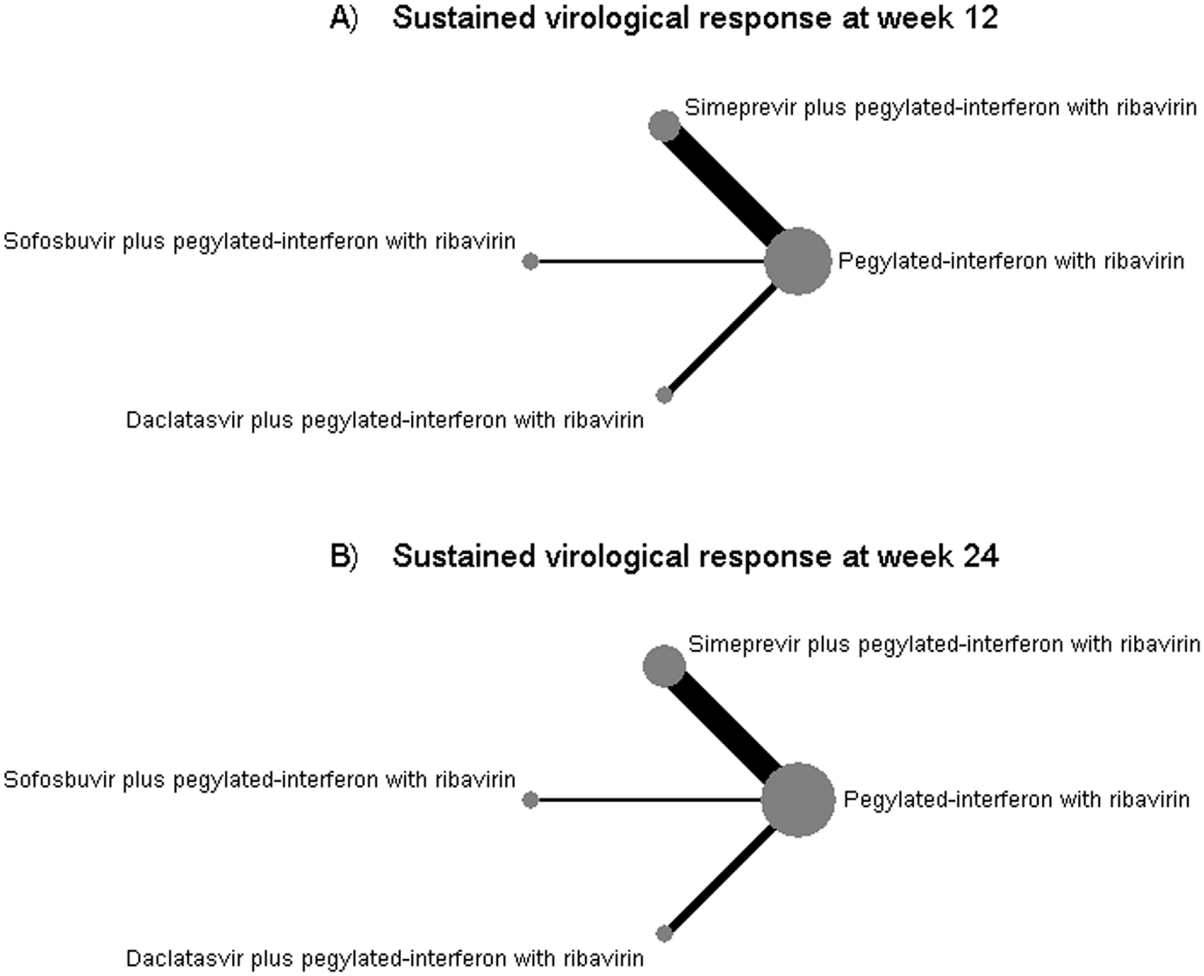
Network plots for sustained virological response at weeks 12 and 24 after the end of treatment.

For indirect comparisons, 8 [4, 11–16, 22] (n = 1,951) and 9 studies [4, 10–16, 22] (n = 1,859) were respectively included in the analyses of SVR12 and SVR24. The number of participants who have SVR12 and SVR24 for each study are presented in S2 Table. Network plot was constructed to map 4 treatment regimens (i.e. SMV plus PR, SOF plus PR, DCV plus PR, and PR alone) where data were available, see Fig 2A and 2B. Size of node and edge respectively reflect number of studies and patients for that comparison; which show that PR was the only common comparator and had the largest sample size among the 4 treatment regimens, whereas the SMV plus PR versus PR had the largest number of studies.

#### Sustained virological response at week 12

The pooled incidence of SVR12 for PR, SMV plus PR, SOF plus PR, and DCV plus PR regimens were respectively 51% (95%CI: 43%, 59%), 83% (95%CI: 79%, 86%), 82% (95%CI: 63%, 100%), and 65% (95%CI: 57%, 73%), see S3 Table. A two-stage multivariate meta-analysis was applied and suggested that the chance of having SVR12 was significantly higher in SMV plus PR and DCV plus PR regimens when compared with PR alone. The pooled RRs for SMV plus PR and DCV plus PR were of 1.48 (95%CI: 1.27, 1.72) and 1.82 (95%CI: 1.24, 2.69), respectively (see Table 3). SOF plus PR regimen also increased SVR12 when compared to PR, but this did not reach statistical significance (pooled RR = 1.52; 95%CI: 0.97, 2.40), see Table 3. Treatment ranking was then assessed by estimating probability of being the best treatment, which yielded probabilities of 65.5%, 28%, and 6.5% for DCV plus PR, SOF plus PR, and SMV plus PR regimens, respectively. This indicated that the best treatment regimen was DCV plus PR followed by SOF plus PR.

**Table 3:** Network meta-analysis of sustained virological response at week 12 after the end of treatment.

Treatment	No. of subjects	No. of SVR12	Pooled RR (95% CI)
**DAA plus PR versus PR alone**			
PR	525	271	1
SMV plus PR	953	780	1.48 (1.27, 1.71)
SOF plus PR	144	121	1.52 (0.97, 2.40)
DCV plus PR	329	209	1.82 (1.24, 2.70)
**Among DAA plus PR regimens**			
SOF plus PR vs SMV plus PR	-	-	1.03 (0.64, 1.66)
DCV plus PR vs SMV plus PR	-	-	1.23 (0.81, 1.87)
DCV plus PR vs SOF plus PR	-	-	1.20 (0.66, 2.18)

CI, confidence interval; DCV, daclatasvir; DAA, direct acting anti-viral agents; LDV, ledipasvir; PR, pegylated interferon-ribavirin; RR, risk ratio; SMV, simeprevir; SOF, sofosbuvir; SVR12, sustained virological response at week 12 after the end of treatment.

**Fig 3:**
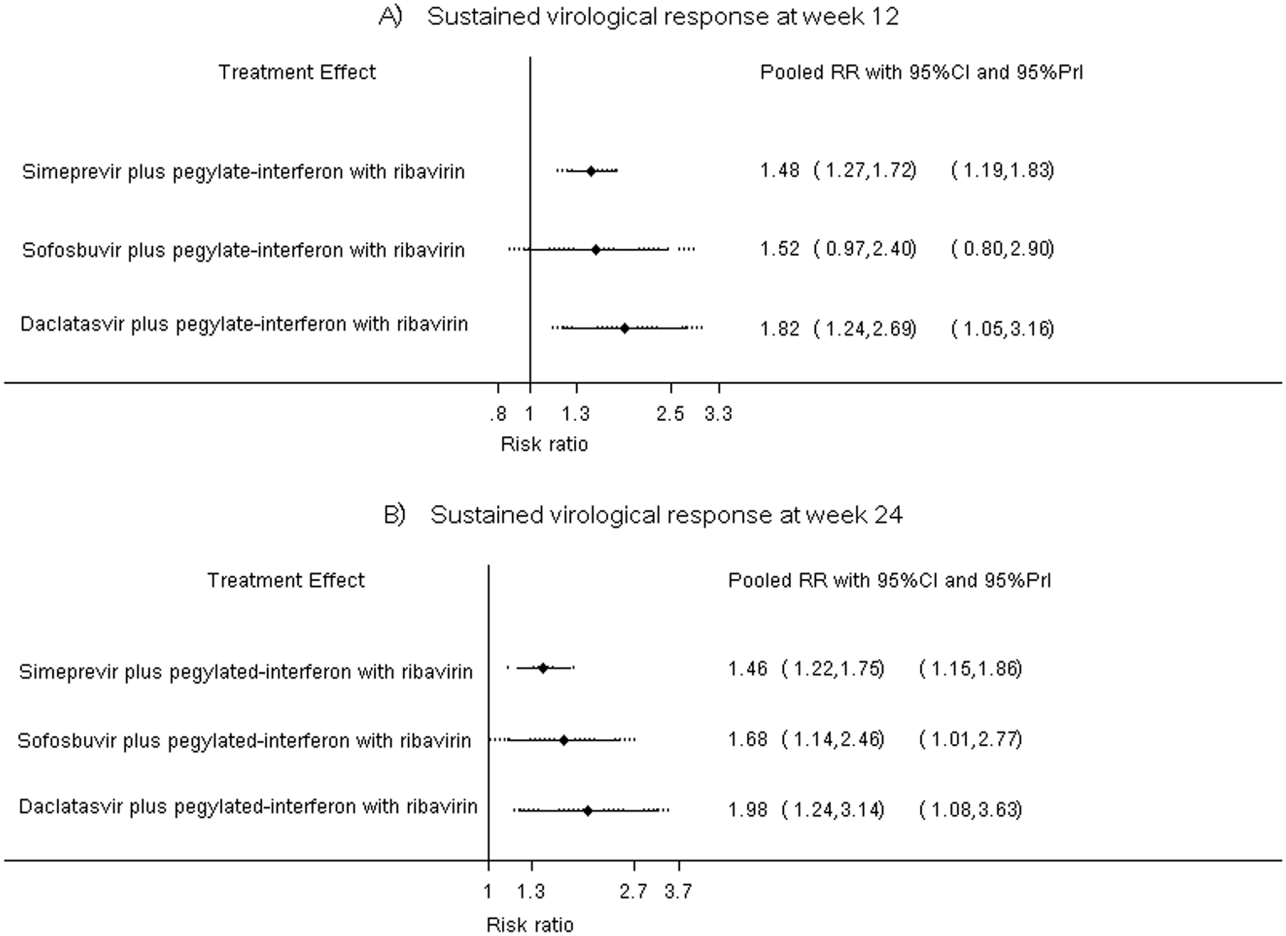
Predictive interval plots for sustained virological response at weeks 12 and 24 after the end of treatment.

The predictive interval was also estimated to predict whether treatment effect will exist in the future by taking into account uncertainty and heterogeneity. The plot indicated that SMV plus PR and DCV plus PR regimens will be effective in the future after accounting for uncertainty and heterogeneity, see Fig 3A. The inconsistency assumption was assessed by estimating inconsistency factors (i.e. difference between direct and indirect lnRR), which were 0.012 (Z = 0.122, P-value = 0.903), 0.001 (Z = 0.003, P-value = 0.998), and 0.010 (Z = 0.024, P-value = 0.981) for SMV plus PR, SOF plus PR, and DCV plus PR, respectively. These suggested that the estimated treatment effects between direct and indirect comparisons were not significantly different.

When comparing among the different DAA plus PR regimens, SVR12 was not significantly different among all treatment comparisons. Pooled RRs of SVR12 between SOF plus PR versus SMV plus PR, DCV plus PR versus SMV plus PR, and DCV plus PR versus SOF plus PR regimens were 1.03 (95%CI: 0.64, 1.66), 1.23 (95%CI: 0.81, 1.87), and 1.20 (95%CI: 0.66, 2.18), respectively (see Table 3).

#### Sustained virological response at week 24

Pooled incidence of SVR24 for PR, SMV plus PR, SOF plus PR, and DCV plus PR regimens were 48% (95%CI: 40%, 57%), 83% (95%CI: 80%, 86%), 81% (95%CI: 68%, 95%), and 62% (95%CI: 53%, 70%), respectively (see S3 Table). When compared with PR alone, all DAA plus PR regimens significantly increased SVR24 with pooled RRs of 1.46 (95%CI: 1.22, 1.75), 1.98 (95%CI 1.24, 3.14), and 1.68 (95%CI: 1.14, 2.46) for SMV plus PR, SOF plus PR, and DCV plus PR regimens, respectively (see Table 4). SOF plus PR regimen had the highest probability for being the best treatment (74.5%), followed by DCV plus PR (24.5%), and SMV plus PR (1%) regimen.

**Table 4:** Network meta-analysis of sustained virological response at week 24 after the end of treatment.

Treatment	No. of subjects	No. of SVR12	Pooled RR (95% CI)
**DAA plus PR versus PR alone**			
PR	375	187	1
SMV plus PR	1011	833	1.46 (1.22, 1.75)
SOF plus PR	144	119	1.98 (1.24, 3.14)
DCV plus PR	329	199	1.68 (1.14, 2.46)
**Among DAA plus PR regimens**			
SOF plus PR vs SMV plus PR	-	-	1.35 (0.82, 2.23)
DCV plus PR vs SMV plus PR	-	-	1.15 (0.75, 1.76)
DCV plus PR vs SOF plus PR	-	-	0.85 (0.46, 1.55)

CI, confidence interval; DCV, daclatasvir; DAA, direct acting anti-viral agents; LDV, ledipasvir; PR, pegylated interferon-ribavirin; RR, risk ratio; SMV, simeprevir; SOF, sofosbuvir; SVR24, sustained virological response at week 24 after the end of treatment.

Fig 3B illustrates the predictive interval plot for SMV plus PR, SOF plus PR, and DCV plus PR, when compared with PR alone. All three DAA plus PR regimens seem to be effective in the future after taking into account both uncertainty and heterogeneity. In addition, the inconsistency factors for SMV plus PR, SOF plus PR, and DCV plus PR were 0.0002 (Z = 0.001, P-value = 0.998), 0.002 (Z = 0.007, P-value = 0.994), and 0.011 (Z = 0.046, P-value = 0.963), respectively. These indicated that results from direct and indirect estimations were consistent for all 3 treatment regimens.

When compared the efficacy among the DAA plus PR regimens, the probability of having SVR24 was not significantly different among these regimens. The pooled RRs for SOF plus PR versus SMV plus PR, DCV plus PR versus SMV plus PR, and DCV plus PR versus SOF plus PR were 1.35 (95%CI: 0.82, 2.23), 1.15 (95%CI: 0.75,1.76), and 0.85 (95%CI: 0.46,1.55), respectively (see Table 4).

### Dual DAA combinations with ribavirin versus dual DAA combinations without ribavirin

Pooled incidence rates of SVR12 for SOF plus DCV, SOF plus LDV, SOF plus SMV, SOF plus DCV with ribavirin, SOF plus LDV with ribavirin, and SOF plus SMV plus ribavirin were 100% (95%CI: 95%,100%), 98% (95%CI: 95%,100%), 97% (95%CI: 90%,100%), 96% (95%CI: 92%,100%), 97% (95%CI: 95%,100%), and 93% (95%CI: 86%,100%), respectively (see S3 Table). Only 4 studies [17–19, 23] that compared SOF plus LDV and ribavirin (N = 754) with SOF plus LDV (N = 984) had sufficient data for performing direct meta-analysis. The probability of having SVR12 in patients receiving the SOF plus LDV with ribavirin regimen was not significantly different from patients receiving SOF plus LDV regimen, with a pooled RR of 0.99 (95% CI: 0.97, 1.01). There was no evidence of heterogeneity of treatment effect between studies with an I^2^ value of 7% (see Table 5 and S2 Fig). The chances of having SVR12 were also not different between SOF plus DCV and ribavirin and SOF plus DCV (RR = 0.96; 95% CI: 0.91, 1.02) as well as SOF plus SMV and ribavirin and SOF plus SMV (RR = 0.96, 0.87, 1.06).

**Table 5:** Pooled risk ratio of sustained virological response at week 12 after the end of treatment between sofosbuvir plus ledipasvir with ribavirin and sofosbuvir plus ledipasvir.

Author	Year	SOF plus LDV with RBV		SOF plus LDV		RR (95% CI)
		Response	Non-response	Response	Non-response	
Afdhal [18]	2014	426	8	423	8	1.00 (0.98, 1.02)
Kowdley [17]	2014	201	15	408	23	0.98 (0.94, 1.02)
Lawitz [19]	2014	21	0	37	2	1.04 (0.94, 1.15)
Mizokami [23]	2015	80	3	83	0	0.96 (0.92, 1.01)
**Pooled RR**						**0.99 (0.97, 1.01)**

CI, confidence interval; LDV, ledipasvir; RR, risk ratio; RBV, ribavirin; SOF, sofosbuvir; SVR24, sustained virological response at week 24 after the end of treatment.

Only two study [21, 24] reported the outcome of SVR24, of which one study compared the efficacy of SOF plus DCV with and without ribavirin (N = 126) and one study compared the efficacy of PrOD with and without ribavirin (N = 119). Pooled incidence rates of SVR24 for SOF plus DCV, PrOD, SOF plus DCV with ribavirin, and PrOD with ribavirin were 96% (95% CI: 88%, 99%), 89% (95% CI: 79%, 95%), 95% (95% CI: 85%, 99%), and 95% (95% CI: 83%, 99%), respectively (see S3 Table). The chances of having SVR24 were not different between SOF plus DCV and ribavirin and SOF plus DCV (RR = 0.99, 95% CI: 0.69, 1.42) as well as PrOD plus ribavirin and PrOD without ribavirin (RR = 1.07, 95% CI: 0.96, 1.19).

### Adverse drug events

#### Serious adverse events

Fourteen studies [4, 10–19, 21, 22, 24] (N = 3,860) provided data about serious adverse events which occurred during the entire treatment. The pooled incidence of serious adverse events for all treatment regimens are presented in S4 Table. Pooled incidence rate of serious adverse drug events in treatment regimens with PR were 7.9% (95% CI: 5.1%, 10.7%), 5.0% (95% CI: 3.6%, 6.3%), 4.10% (95% CI: 0.0%, 9.6%) and 7.5% (95% CI: 5.2%, 9.7%) for DCV plus PR, SMV plus PR, SOF plus PR and PR alone, respectively. Pooled incidence rates of serious adverse events in all regimens without PR were lower than the pooled incidence rates in all regimens with PR, except for SOF plus DCV regimen (pooled incidence = 7.0% (95% CI: 2.0%, 12.0%)). Incidence rate was lowest in SOF plus LDV with ribavirin regimen (1.9%; 95% CI: 0%, 4.5%), followed by PrOD (2.5%; 95% CI: 0.3%, 8.8%), PrOD with ribavirin (2.8%; 95% CI: 0.1%, 14.5%), SOF plus DCV with ribavirin (2.9%; 95% CI: 1.0%, 6.8%), and SOF plus LDV (3.0%; 95% CI: 1.3,% 4.7%).

#### Anemia

The pooled incidence rates of anemia from 13 studies [4, 10–19, 22, 24] (N = 3,764) are presented in S5 Table. SOF plus LDV regimen had the lowest incidence rate of anemia (0.9%; 95% CI: 0.0%, 1.8%) and all treatment regimens without PR had lower incidence rates of anemia than all regimens with PR. The highest incidence rate was found in SMV plus PR regimen with pooled incidence rate of 29.1% (95% CI: 18.6%, 39.6%).

#### Fatigue

The pooled incidence rates of fatigue for each treatment regimen from 12 studies [4, 10, 11, 13–18, 21, 22, 24] (N = 3,672) are presented in S6 Table. Nearly half of patients receiving treatment regimens with PR had fatigue with the highest incidence rate in SOF plus PR regimen (56.7%; 95% CI: 34.8%, 78.7%), followed by DCV plus PR (54.7%; 95% CI: 49.5, 59.9), PR (50.0%; 95% CI: 42.9%, 57.1%), and SMV plus PR (44.8%; 95% CI: 37.7%, 51.9%). Patients receiving treatment regimens without PR had a lower rate of fatigue than patients receiving treatment regimens with PR.

### Publication bias

Publication bias was assessed for outcome of SVR12. Results from the Egger test suggested no publication bias for both SMV plus PR versus PR regimen (coefficient = -0.12, P-value = 0.666) and SOF plus LDV with ribavirin versus SOF plus LDV regimen (coefficient = -0.001, P-value = 0.797). A funnel plot also showed no small study effect for both SMV plus PR versus PR regimen (see S3 Fig) and SOF plus LDV with ribavirin versus SOF plus LDV regimen (see S4 Fig).

## Discussion

Our study showed that all DAA plus PR regimens are superior to PR alone. Among them, SOF plus PR is the best treatment regimen that could achieve SVR24, followed by DCV plus PR. However, there is no significant difference of treatment efficacy among DAA plus PR regimens. In addition, adding ribavirin to the dual DAA regimens showed no significant difference compared to the dual DAAs without ribavirin. Regarding adverse reactions, treatment regimens with PR had a relatively higher risk of serious adverse drug events, anemia and fatigue, than the treatment regimens without PR.

The above findings are in line with previous systematic reviews [25, 26] that recommended DAA plus PR regimens, especially SOF plus PR for the first-line drug of treatment naïve HCV *genotype 1*. However, the previous studies did not apply network meta-analysis in order to address the efficacy between the different DAA plus PR regimens and PR alone, and this was rectified in this study. However, our review did not compare treatment regimens without PR (i.e. dual DAAs with/without ribavirin) with PR alone. Pooled incidence rates of SVR12 and SVR24 in all treatment regimens without PR (93% to 100% for SVR12, and 89% to 96% for SVR24) were much higher than the incidence rate of SVR12 and SVR24 in PR alone (51% for SVR12 and 48% for SVR24). In addition, the risk of having adverse events (i.e. serious adverse events, anemia and fatigue) were much lower in treatment regimens without PR than treatment regimens with PR. These findings were corresponded with the results from Bansal et al.’s study, in which combining two DAAs increased chance of having SVR24 (pooled SVR24 = 96.4%, 95% CI: 93.6%, 98.0%) with a low risk of serious adverse events (pooled incidence of serious adverse events = 1.9%, 95% CI: 0.6%, 5.7%) [27]. Therefore, dual DAA with/without ribavirin regimens might be added in the first-line treatment of HCV *genotype 1*.

For the first generation of DAA (i.e. telaprevir, boceprevir), previous meta-analysis suggested that these drugs could improve chance of having SVR in treatment naïve HCV genotype 1. However, both telaprevir and boceprevir significantly increased risk of adverse drug events (e.g. anemia and rash) and have an issue of pill burden [28]. Because of these reasons, we did not include the first generation DAA in our review.

This study also found that the chance of having SVR12 corresponded with the chance of having SVR24 in all treatment regimens. Therefore, measuring SVR at week 24 after treatment offers lesser benefit compared to measuring at week 12, which would save costs for future Hepatitis C studies and treatment if applied.

### Role of interferon therapy in the future

Interferon has been the standard treatment for HCV for over 20 years. However, the role of interferon based regimen might become less important in the near future due to the clearly benefit of interferon free regimens (i.e. two DAAs with or without ribavirin) over interferon based regimens. Nevertheless, due to the very high cost of DAA (cost of SOF = 84,000 U.S. dollars/course, cost of SOF plus LDV = 94,500 U.S. dollars/course), interferon based therapy has still been the preferable option for standard treatment of HCV infected patients, especially in the low and middle income countries or paying the treatment out-of-pocket. Apart from the cost, interferon also has a therapeutic role in patients with favorable interferon-response characteristics, particularly a favorable IL28B genotype. Evidence from genome-wide association studies suggests that single-nucleotide polymorphisms near the IL28B gene are significantly associated with response to interferon based therapy [29]. Therefore, using interferon based regimens should be considered in the regions with high prevalence of favorable IL28B genotype such as Asia [30].

### Strengths and limitations of the study

Our review has several strengths. We performed a comprehensive search for identifying relevant studies and included all possible regimens for treatment naïve HCV *genotype 1*. All important outcomes were considered including SVR12, SVR24, and adverse drug events. However, our study does have some limitations. Firstly, most of the eligible studies (12 of 16) included only non-cirrhotic patients. Thus, the results from our reviews may not be applicable for the whole spectrum of treatment naïve HCV *genotype 1* patients. Moreover, in some studies, similar interventions were divided into several arms which were different in dosages and duration of treatment. For our analysis, we combined these arms into a single treatment and this may be the cause of heterogeneity in our study. In addition, we could not perform a subgroup analysis according to dosages and duration of treatment due to the small number of studies. Therefore, the dose and duration of recommended treatment have been suggested in range and not in definite value.

### Further study

The new DAAs are much more expensive than PR. For instance, the cost of SOF and SOF plus LDV for a 12-week treatment course is 84,000 U.S. dollars [31] and 94,500 U.S dollars, respectively, while PR for a 48-week treatment course is only 28,444 U.S. dollars [32]. However, findings from our study revealed that DAA plus PR and dual DAA regimens had much higher SVR and lower side effects than PR alone. Moreover, the course of treatment is also shorter and more convenient. Therefore, the cost-effectiveness of the DAA plus PR and dual DAA regimens needs to be analyzed. Information from an economic evaluation will be very useful for policy decision-making for the reimbursement of treatment naïve HCV *genotype 1* treatment, especially in low- and middle-income countries.

### Conclusion

All DAA plus PR regimens are superior to PR alone. Dual DAA regimens also had a higher chance of having SVR12 and SVR24 and had a lower risk of developing adverse events than PR alone. Therefore, both DAA plus PR and dual DAA regimens should be included as first line drugs for treatment naïve HCV *genotype 1*. However, due to high drug costs, an economic evaluation should be conducted in order to assess the value of the investment when making coverage decisions.

## Supporting Information

S1 Appendix Search terms for Medline. (PDF)

S2 Appendix Search terms for Scopus. (PDF)

S1 Checklist PRISMA checklist. (PDF)

S1 Fig Forest plot of pooled risk ratio for comparison between simeprevir plus pegylated-interferon with ribavirin and pegylated-interferon with ribavirin alone. (PDF)

S2 Fig Forest plot of pooled risk ratio of sustained virological response at week 12 for comparison between sofosbuvir plus ledipasvir with ribavin and sofosbuvir plus ledipasvir. (PDF)

S3 Fig Funnel plot of risk ratios of sustained virological response at 12 weeks after the end of treatment between simeprevir plus pegylated interferon-ribavirin and pegylated interferon-ribavirin alone. (PDF)

S4 Fig Funnel plot of risk ratios of sustained virological response at week 12 after the end of treatment between sofosbuvir-ledipasvir with ribavirin and sofosbuvir-ledipasvir. (PDF)

S1 Protocol Study’s protocol. (PDF)

S1 Table Risk of bias assessment of included studies. (PDF)

S2 Table Frequencies of patients who have sustained virological response at weeks 12 and 24 after the end of treatment for treatment regimens included in network meta-analysis. (PDF)

S3 Table Pooled incidence rate of sustained virological response at weeks 12 and 24 after the end of treatment. (PDF)

S4 Table Pooled incidence rate of serious adverse event at entire of treatment. (PDF)

S5 Table Pooled incidence rate of anemia at entire of treatment. (PDF)

S6 Table Pooled incidence rate of fatigue at entire of treatment. (PDF)
