# Supplementary material for: Efficacy of Second Generation Direct-Acting Antiviral Agents for Treatment Naïve Hepatitis C Genotype 1: A Systematic Review and Network Meta-Analysis
Source: PLoS One. 2015 Dec 31;10(12):e0145953. doi: 10.1371/journal.pone.0145953 (PMC4701000; doi:10.1371/journal.pone.0145953)
Supplement: S1 Appendix — (PDF) [file pone.0145953.s001.pdf]

Appendix A. Search terms for Medline

((((((HCV) OR "hepatitis c") OR genotype)) AND (((((((((sofosbuvir) OR Solvadi) OR simeprevir) OR Olysio) OR ledipasvir) OR Harvoni) OR "Viekira Pak") OR ((sofosbuvir) AND daclatasvir)) OR ((((Ombitasvir) AND Paritaprevir) AND Ritonavir) AND Dasabuvir)))) AND ((randomized) OR randomised)
