# Supplementary material for: Efficacy of Second Generation Direct-Acting Antiviral Agents for Treatment Naïve Hepatitis C Genotype 1: A Systematic Review and Network Meta-Analysis
Source: PLoS One. 2015 Dec 31;10(12):e0145953. doi: 10.1371/journal.pone.0145953 (PMC4701000; doi:10.1371/journal.pone.0145953)
Supplement: S2 Appendix — (PDF) [file pone.0145953.s002.pdf]

Appendix B. Search terms for Scopus

((TITLE-ABS-KEY (HCV)) OR (TITLE-ABS-KEY ("hepatitis C")) OR OR (TITLE-ABS-KEY (genotype))) AND ((TITLE-ABS-KEY (Solvadi)) OR (TITLE-ABS-KEY (sofosbuvir)) OR (TITLE-ABS-KEY (simeprevir)) OR (TITLE-ABS-KEY (Olysio)) OR (TITLE-ABS-KEY (ledipasvir)) OR (TITLE-ABS-KEY (Harvoni)) OR (TITLE-ABS-KEY (“Viekira Pak”)) OR (TITLE-ABS-KEY (Ombitasvir AND Paritaprevir AND Ritonavir)) OR (TITLE-ABS-KEY (daclatasvir)))
