# Supplementary material for: Efficacy of Second Generation Direct-Acting Antiviral Agents for Treatment Naïve Hepatitis C Genotype 1: A Systematic Review and Network Meta-Analysis
Source: PLoS One. 2015 Dec 31;10(12):e0145953. doi: 10.1371/journal.pone.0145953 (PMC4701000; doi:10.1371/journal.pone.0145953)

**S1 Figure. Forest plot of pooled risk ratio for comparison between simeprevir plus pegylated-interferon with ribavirin and pegylated-interferon with ribavirin alone**

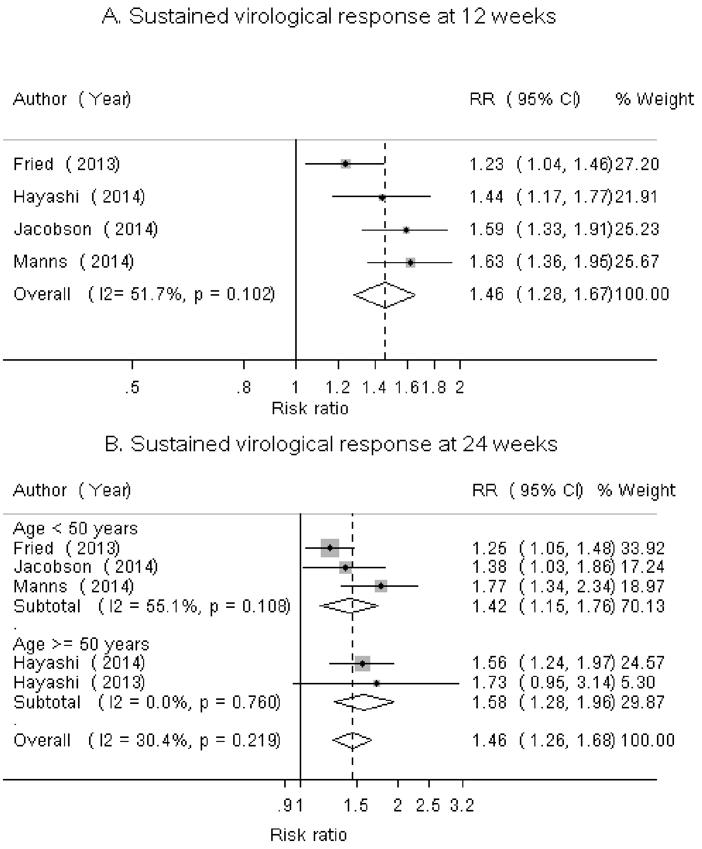

Supplement: S1 Fig — (PDF) [file pone.0145953.s004.pdf]
