# Supplementary material for: Efficacy of Second Generation Direct-Acting Antiviral Agents for Treatment Naïve Hepatitis C Genotype 1: A Systematic Review and Network Meta-Analysis
Source: PLoS One. 2015 Dec 31;10(12):e0145953. doi: 10.1371/journal.pone.0145953 (PMC4701000; doi:10.1371/journal.pone.0145953)

S2 Figure. Forest plot of pooled risk ratio of sustained virological response at week 12 for comparison between sofosbuvir plus ledipasvir with ribavin and sofosbuvir plus ledipasvir

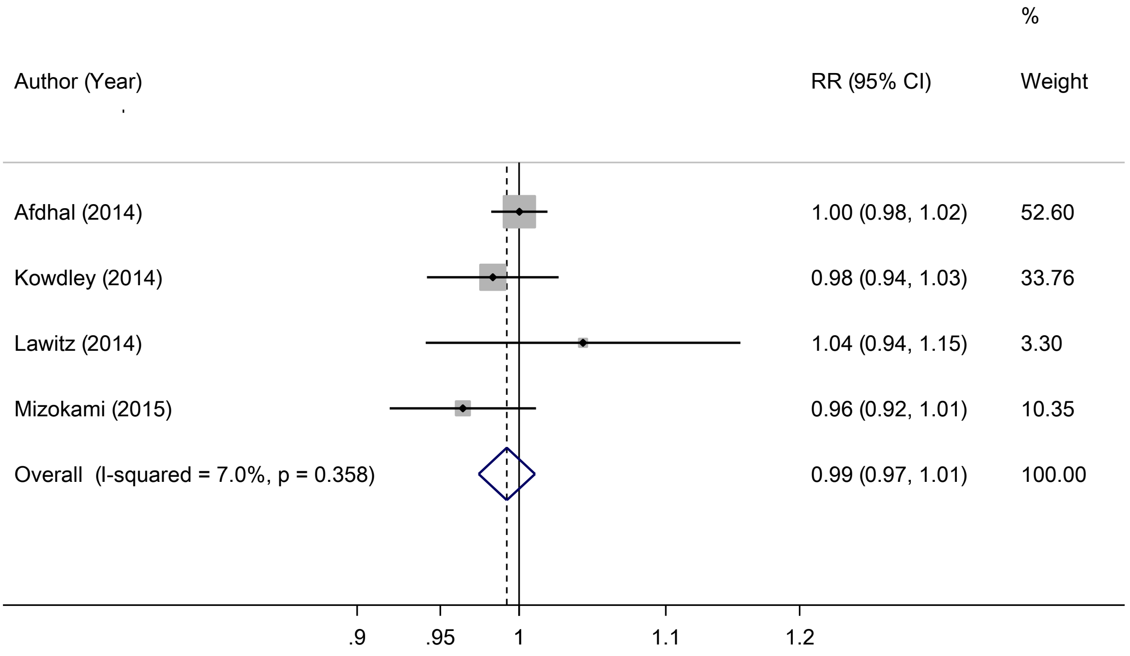

Supplement: S2 Fig — (PDF) [file pone.0145953.s005.pdf]
