# Supplementary material for: Efficacy of Second Generation Direct-Acting Antiviral Agents for Treatment Naïve Hepatitis C Genotype 1: A Systematic Review and Network Meta-Analysis
Source: PLoS One. 2015 Dec 31;10(12):e0145953. doi: 10.1371/journal.pone.0145953 (PMC4701000; doi:10.1371/journal.pone.0145953)

S3 Figure. Funnel plot of risk ratios of sustained virological response at 12 weeks after the end of treatment between simeprevir plus pegylated interferon-ribavirin and pegylated interferon-ribavirin alone

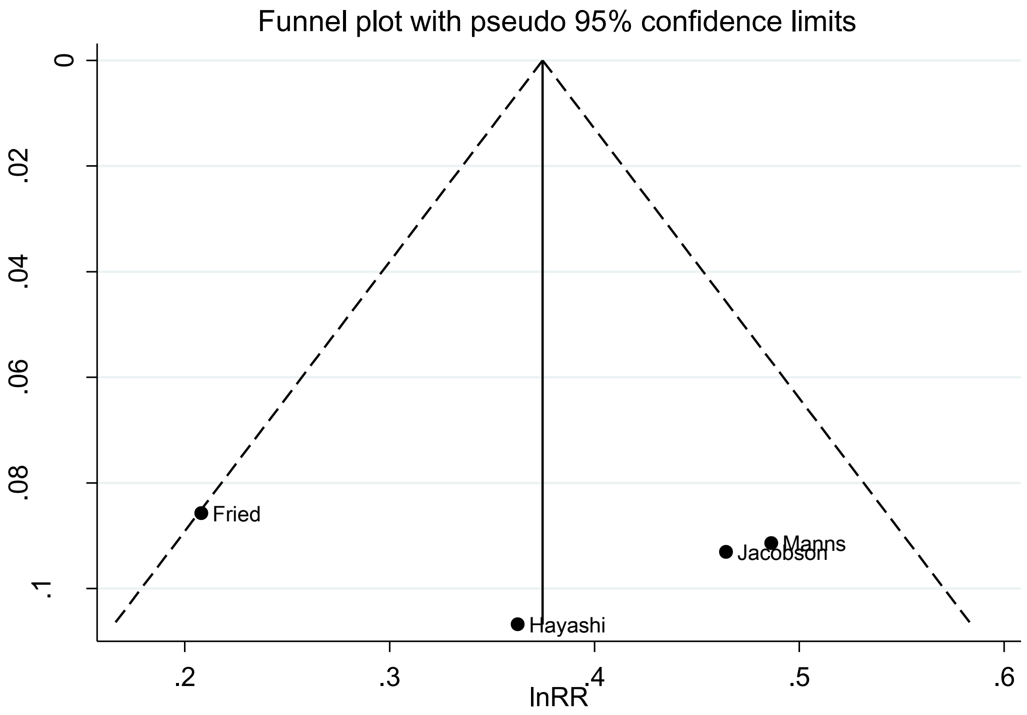

Supplement: S3 Fig — (PDF) [file pone.0145953.s006.pdf]
