# Supplementary material for: Efficacy of Second Generation Direct-Acting Antiviral Agents for Treatment Naïve Hepatitis C Genotype 1: A Systematic Review and Network Meta-Analysis
Source: PLoS One. 2015 Dec 31;10(12):e0145953. doi: 10.1371/journal.pone.0145953 (PMC4701000; doi:10.1371/journal.pone.0145953)

S4 Figure. Funnel plot of risk ratios of sustained virological response at week 12 after the end of treatment between sofosbuvir-ledipasvir with ribavirin and sofosbuvir-ledipasvir

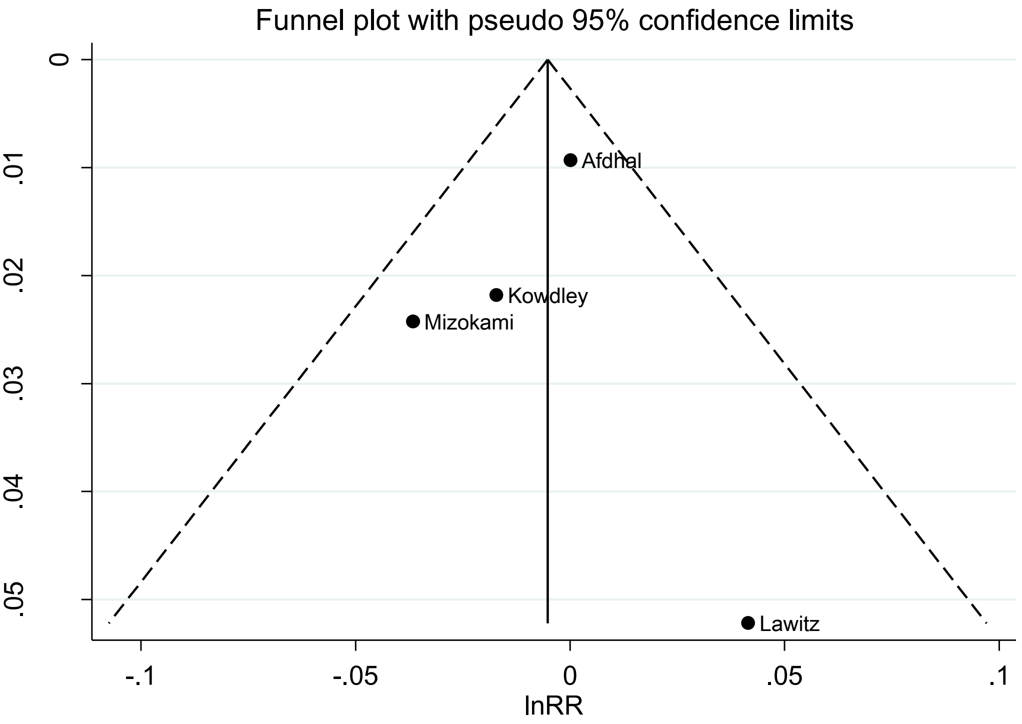

Supplement: S4 Fig — (PDF) [file pone.0145953.s007.pdf]
