# Supplementary material for: Efficacy of Second Generation Direct-Acting Antiviral Agents for Treatment Naïve Hepatitis C Genotype 1: A Systematic Review and Network Meta-Analysis
Source: PLoS One. 2015 Dec 31;10(12):e0145953. doi: 10.1371/journal.pone.0145953 (PMC4701000; doi:10.1371/journal.pone.0145953)
Supplement: S1 Protocol — (PDF) [file pone.0145953.s008.pdf]

Efficacy of interferon based and interferon free regimens for treatment naïve chronic hepatitis C genotype 1: A systematic review and network meta-analysis

Hepatitis C virus (HCV) infection is the major public health problem. Approximately 2-3% of the world's population have been infected with HCV and 3-4 million new cases of HCV infection have been reported every year [1]. HCV infection is the significant cause of cirrhosis, hepatocellular carcinoma, and liver transplantation [2-4]. HCV has 6 clinically main genotypes, of which genotype 1 is the most prevalent worldwide, especially in the United States and European countries [5,6]. In the past decade, standard treatment for all HCV genotypes is combination of pegylated-interferon with ribavirin(PR). However, PR has limitation for treatment of HCV genotype 1 due to suboptimal sustained virological response (SVR). Only 45% of patients with genotype1 could achieve SVR after 48 weeks of PR treatment [7,8] and around 9-15% of patients could not tolerate to PR due to several side effects such as flu-like symptoms, anemia, neutropenia, thrombocytopenia, and depression [9]. Therefore, more effective and tolerable treatment regimens are required for HCV genotype1.

Direct-acting antiviral agents (DAAs), specifically targeting on HCV protein, has been developed over the past 10-15 years. DAAs have been divided into 3 major groups (i.e. NS3/4A protease inhibitors, NS5A inhibitors, and NS5B inhibitors), of which, telaprevir and boceprevir are the first licensed DAAs and when combined with PR can improve the SVR in HCV genotype 1. However, telaprevir and boceprevir have complex dosing regimens and are associated with several adverse drug events (e.g. anemia and rash). Simeprevir (SMV), second generation of NS3/4A protease inhibitors, can achieve a higher rate of SVR (80-90%) for treatment of HCV genotype 1, than treating with PR alone (SVR rate = 45%). Sofosbuvir (SOF), the first licensed drug in NS5B inhibitors group, and daclatasvir (DCV) in NS5A inhibitors group, combined with PR, respectively increase SVR rate as high as 90% [10-12] and 43-87%[13] for treatment naïve HCV genotype 1.

Even though DAAs plus PR could achieve high SVR in treatment naïve HCV genotype 1. The effective interferon-free treatment is also needed to avoid the need for subcutaneous injections and the side effects from the use of interferon. Results from LONSESTAR trial found that combination of sofosbuvir and ledipasvir (LDV) with or without ribavirin had SVR of 95-100% in treatment naïve HCV genotype 1 [14]. In addition giving SOF plus DCV with or without ribavirin for treatment naïve HCV genotype 1 achieved the high SVR of 94-98% [15]. Although the interferon free regimens

(combination of DAADs with or without ribavirin) showed the promising results. There has been no randomized-controlled trial that directly compare the efficacy of interferon based regimens (DAAs plus PR, or PR alone) with interferon free regimens (dual DAA combinations with or without ribavirin). Therefore, we will perform a systematic review and network meta-analysis aimed to assess the efficacy of all possible DAA regimens when compared with PR in treatment naïve hepatitis C genotype 1.

Objectives of the review

1. To estimate the pooled incidence rate of sustained virological response at weeks 12 and 24 after the end of treatment in DAA plus PR, dual DAA with/without ribavirin, and PR regimens.
2. To estimate the pooled risk ratios of sustained virological response at weeks 12 and 24 between DAA plus PR regimens and PR alone.
3. To estimate the pooled risk ratios of sustained virological response at weeks 12 and 24 among different DAA plus PR regimens.
4. To estimate the pool the risk ratios of sustained virological response at weeks 12 and 24 between dual DAA regimens with/without ribavirin and PR alone.
5. To estimate the pooled risk ratios of sustained virological response at weeks 12 and 24 among different dual DAA regimes.
6. To estimate the pooled incidence of adverse drug events in DAA plus PR, dual DAA, and PR regimens.

Methods

Literature search

The relevant studies will be searched from Medline and Scopus databases since inception up to 25th May 2015. Reference lists of eligible studies and previous systematic reviews will be also explored for identifying additional relevant studies. Search terms for both Medline and Scopus database will be as follows.

- HCV
- "hepatitis c"
- Genotype

- Sofosbuvir
- Solvadi
- Simeprevir
- Olysio
- Ledipasvir
- Harvoni
- "Viekira Pak"
- Daclatasvir
- Ombitasvir
- Paritaprevir
- Ritonavir
- Dasabuvir

Study selection

Relevant studies will be independently selected based on titles and abstracts by two reviewers. Full articles will be ascertained if the decision cannot be made based on titles and abstracts. Disagreement between the two reviewers will be decided by consensus with a third party.

Inclusion criteria

Randomized controlled trials published in English will be selected if they meet all of the following criteria.

1. Study's participants were treatment naïve HCV genotype 1
2. The study compared the efficacy of any pairs of the following regimens: DAA plus PR, dual DAA combinations with and without ribavirin, or PR alone.
3. Study measured the outcomes as SVR at weeks 12 or 24 after the end of treatment
4. Study reported the number of patients having or not having SVR in each treatment regimen.

The Studies will be excluded if they only compared the efficacy between different dosages of the same treatment regimens.

Data extraction

Two reviewers will independently extract the data. These following data will be extracted.

- The baseline characteristics of the eligible studies including
    - Author's names, year of publication, mean age, body mass index (BMI), and baseline HCV RNA of the study's participants, percentages of sex, and cirrhosis
- The outcomes of interest including
    - Total number of patients who had SVR at weeks 12 and 24 after the end of treatment, and adverse drug events between treatment and outcome groups.

The corresponding authors of the included studies will be contacted if there have any missing or inadequate information.

Treatment regimens of interest

Due to lower efficacy and more side effects of first generation DAAs, this review will include only second generation DAAs approved by the US or EU Food and Drug, comprising simeprevir (SMV), sofosbuvir (SOF), daclatasvir (DCV), ledipasvir (LDV), and paritaprevir/ritonavir/ombitasvir plus dasabuvir (Viekira Pak).

Treatment regimens of interest will be divided into DAA plus PR regimens (i.e. SMV plus PR, DCV plus PR, and SOF plus PR), dual DAA combinations with and without ribavirin regimens (i.e. SOF plus LDV, SOF plus SMV, SOF plus DCV, SOF plus LDV and ribavirin, SOF plus SMV and ribavirin, and SOF plus DCV and ribavirin, Viekira Pak and Viekira Pak plus ribavirin), and PR alone.

Outcomes

SVRs at weeks 12 (SVR12) and 24 (SVR24) after the end of treatment are the outcome of interest. SVR12 and SVR24 are defined as HCV RNA levels lower than the detectable level specified in the eligible studies at weeks 12 and 24 after the end of treatment. Safety outcomes considered in this review are anemia, fatigue, and serious adverse drug events, defined as death or serious conditions which required hospital admission.

Risk of bias assessment

Risk of bias assessment will be performed was using the Cochrane Collaboration's tool for assessing risk of bias in randomized controlled trials [16]. Two reviewers will independently perform

the risk of bias assessment. Disagreement between two reviewers will be resolved by consensus with a third party.

Statistical analysis

1. *Pooling incidence rate of SVR12, SVR24, and adverse drug events*

Incidence rate of adverse drug events and SVR12 and SVR24 for each treatment regimen will be pooled using ‘pmeta’ command in STATA program.

2. *Direct meta-analysis*

A direct meta-analysis will be performed for studies that have similar treatment comparisons if a total number of the studies is not less than 3 studies. Risk ratios (RR) of SVR12 and SVR24 will be estimated for each study and then will be pooled using the inverse variance method if there is no heterogeneity between studies; otherwise, a random effect model will be applied. Q test and I^2 statistic will be applied for assessing the heterogeneity between studies. Heterogeneity between studies will be presented, if the degree of heterogeneity (I^2) is higher than 25%. Sources of heterogeneity will be explored by fitting co-variables (i.e. mean age, BMI, baseline HCV RNA, and percent cirrhosis) one by one in a meta-regression, if the heterogeneity is present.

3. *Indirect meta-analysis*

For indirect comparisons, treatment effects of all treatment regimens will be estimated by applying a two-stage network meta-analysis. First, summary data will be expanded into individual patient data using ‘expand’ command in STATA. Poisson regression will be applied to estimate log (RR) and variance-covariance of each treatment pairwise using mvmeta_make command. Pooled RRs and their 95% confidence interval (CI) will be estimated using a multivariate random effect meta-analysis, in which within subject-study correlation will be accounted for using Riley’ method. Treatment ranking will be evaluated by ranking the linear predictor of each study. For predicting the treatment effect in the future, predictive interval will be estimated by taking into account for uncertainty of the whole network using the ‘intervalplot’ command. The inconsistency assumption (disagreement between direct and indirect estimations) will be tested by measuring the inconsistency factor (difference between lnRRs estimated from direct meta-analysis and indirect meta-analysis).

4. *Publication bias*

Publication bias will be assessed using Egger test and Funnel plot. Publication bias will be presented, if the P-value from Egger test is less than 0.05, or the funnel plot shows asymmetry.

All analyses will performed using STATA version 14.0. A two-sided P value less than 0.05 will be considered statistically significant for all analyses except for heterogeneity test, in which a one-sided P value less than 0.1 will be applied instead.

References

1. Center for Disease Control and Prevention (2014) CDC Health Information for International Travel 2014. Oxford University Press.
2. Bezemer G, Van Gool AR, Verheij-Hart E, Hansen BE, Lurie Y, et al. (2012) Long-term effects of treatment and response in patients with chronic hepatitis C on quality of life. An international, multicenter, randomized, controlled study. BMC Gastroenterol 12: 11.
3. Freeman AJ, Dore GJ, Law MG, Thorpe M, Von Overbeck J, et al. (2001) Estimating progression to cirrhosis in chronic hepatitis C virus infection. Hepatology 34: 809-816.
4. Davis GL, Alter MJ, El-Serag H, Poynard T, Jennings LW (2010) Aging of hepatitis C virus (HCV)-infected persons in the United States: a multiple cohort model of HCV prevalence and disease progression. Gastroenterology 138: 513-521, 521.e511-516.
5. Sievert W, Altraif I, Razavi HA, Abdo A, Ahmed EA, et al. (2011) A systematic review of hepatitis C virus epidemiology in Asia, Australia and Egypt. Liver Int 31 Suppl 2: 61-80.
6. Cornberg M, Razavi HA, Alberti A, Bernasconi E, Buti M, et al. (2011) A systematic review of hepatitis C virus epidemiology in Europe, Canada and Israel. Liver Int 31 Suppl 2: 30-60.
7. Ghany MG, Strader DB, Thomas DL, Seeff LB (2009) Diagnosis, management, and treatment of hepatitis C: an update. Hepatology 49: 1335-1374.
8. Manns MP, McHutchison JG, Gordon SC, Rustgi VK, Shiffman M, et al. (2001) Peginterferon alfa-2b plus ribavirin compared with interferon alfa-2b plus ribavirin for initial treatment of chronic hepatitis C: a randomised trial. Lancet 358: 958-965.
9. McHutchison JG, Lawitz EJ, Shiffman ML, Muir AJ, Galler GW, et al. (2009) Peginterferon alfa-2b or alfa-2a with ribavirin for treatment of hepatitis C infection. N Engl J Med 361: 580-593.
10. Lawitz E, Lalezari JP, Hassanein T, Kowdley KV, Poordad FF, et al. (2013) Sofosbuvir in combination with peginterferon alfa-2a and ribavirin for non-cirrhotic, treatment-naive patients with genotypes 1, 2, and 3 hepatitis C infection: A randomised, double-blind, phase 2 trial. The Lancet Infectious Diseases 13: 401-408.
11. Kowdley KV, Lawitz E, Crespo I, Hassanein T, Davis MN, et al. (2013) Sofosbuvir with pegylated interferon alfa-2a and ribavirin for treatment-naive patients with hepatitis C genotype-1 infection (ATOMIC): an open-label, randomised, multicentre phase 2 trial. Lancet 381: 2100-2107.

12. Lawitz E, Gane EJ (2013) Sofosbuvir for previously untreated chronic hepatitis C infection. N Engl J Med 369: 678-679.
13. Pol S, Ghalib RH, Rustgi VK, Martorell C, Everson GT, et al. (2012) Daclatasvir for previously untreated chronic hepatitis C genotype-1 infection: a randomised, parallel-group, double-blind, placebo-controlled, dose-finding, phase 2a trial. Lancet Infect Dis 12: 671-677.
14. Lawitz E, Poordad FF, Pang PS, Hyland RH, Ding X, et al. (2014) Sofosbuvir and ledipasvir fixed-dose combination with and without ribavirin in treatment-naive and previously treated patients with genotype 1 hepatitis C virus infection (LONESTAR): An open-label, randomised, phase 2 trial. The Lancet 383: 515-523.
15. Sulkowski MS, Gardiner DF, Rodriguez-Torres M, Reddy KR, Hassanein T, et al. (2014) Daclatasvir plus sofosbuvir for previously treated or untreated chronic HCV infection. New England Journal of Medicine 370: 211-221.
16. Higgins JPT, Altman DG, Gøtzsche PC, Jüni P, Moher D, et al. (2011) The Cochrane Collaboration's tool for assessing risk of bias in randomised trials.
