# Supplementary material for: Efficacy of Second Generation Direct-Acting Antiviral Agents for Treatment Naïve Hepatitis C Genotype 1: A Systematic Review and Network Meta-Analysis
Source: PLoS One. 2015 Dec 31;10(12):e0145953. doi: 10.1371/journal.pone.0145953 (PMC4701000; doi:10.1371/journal.pone.0145953)
Supplement: S1 Table — (PDF) [file pone.0145953.s009.pdf]

S1 Table. Risk of bias assessment of included studies

Author	Year	Selection bias		Performance bias	Detection bias	Attrition bias	Reporting bias	Other bias
		Random sequence generation	Allocation concealment	Blinding of participants or personnel	Blinding of outcome assessment	Incomplete outcome data	Selective outcome reporting	
Afdhal[18]	2014	unclear	unclear	low	low	low	low	low
Fried[11]	2013	low	low	low	low	low	low	low
Hayashi[12]	2014	low	low	low	low	low	low	low
Hayashi[10]	2014	unclear	unclear	low	low	low	low	low
Hézode[16]	2014	low	low	low	low	low	low	low
Jacobson[13]	2014	low	low	low	low	low	low	low
Kowdley[17]	2014	unclear	unclear	low	low	low	low	low
Lawitz[19]	2014	low	low	low	low	low	low	low
Lawitz[20]	2014	low	low	low	low	low	low	low
Lawitz[4]	2013	low	low	low	low	low	low	low
Manns[14]	2014	low	low	low	low	low	low	low
Pol[15]	2012	low	low	low	low	high	low	low
Rodriguez-Torres[22]	2013	low	low	low	low	low	low	low
Sulkowski[21]	2014	unclear	unclear	low	low	high	low	low
Kowdley[24]	2014	low	low	low	low	low	low	low
Mizokami[23]	2015	low	low	low	low	low	low	low
