# Supplementary material for: Efficacy of Second Generation Direct-Acting Antiviral Agents for Treatment Naïve Hepatitis C Genotype 1: A Systematic Review and Network Meta-Analysis
Source: PLoS One. 2015 Dec 31;10(12):e0145953. doi: 10.1371/journal.pone.0145953 (PMC4701000; doi:10.1371/journal.pone.0145953)
Supplement: S2 Table — (PDF) [file pone.0145953.s010.pdf]

S2 Table. Frequencies of patients who have sustained virological response at weeks 12 and 24 after the end of treatment for treatment regimens included in network meta-analysis

Author	Year	Treatment	No. of having SVR12	No. of not having SVR12	No. of having SVR24	No. of not having SVR24
Pol[7]	2012	DCV plus PR	26	10	25	11
		PR	3	9	3	9
Rodriguez-Torres[22]	2013	SOF plus PR	35	14	36	13
		PR	7	7	6	8
Lawitz[4]	2013	SOF plus PR	86	9	83	12
		PR	15	11	15	21
Hayashi[10]	2013	SMV plus PR	-	-	63	16
		PR	-	-	6	7
Fried[11]	2013	SMV plus PR	252	57	250	59
		PR	51	26	50	27
Hayashi[12]	2014	SMV plus PR	109	14	109	14
		PR	37	23	34	26
Jacobson[13]	2014	SMV plus PR	210	54	205	42
		PR	65	65	18	12
Manns[14]	2014	SMV plus PR	209	48	206	47
		PR	67	67	28	33
Hezode[16]	2014	DCV plus PR	183	110	174	119
		PR	26	46	27	45

DCV, daclatasvir; LDV, ledipasvir; PR, pegylated interferon-ribavirin; SMV, simeprevir; SOF, sofosbuvir; SVR24, sustained virological response at 24 weeks after the end of treatment
