# Supplementary material for: Efficacy of Second Generation Direct-Acting Antiviral Agents for Treatment Naïve Hepatitis C Genotype 1: A Systematic Review and Network Meta-Analysis
Source: PLoS One. 2015 Dec 31;10(12):e0145953. doi: 10.1371/journal.pone.0145953 (PMC4701000; doi:10.1371/journal.pone.0145953)
Supplement: S3 Table — (PDF) [file pone.0145953.s011.pdf]

**S3 Table. Pooled incidence rate of sustained virological response at weeks 12 and 24 after the end of treatment**

| Treatment | Pooled SVR12 (95% CI) | Pooled SVR24 (95% CI) |
|---|---|---|
| PR | 51% (43%, 59%) | 48% (40%, 57%) |
| SMV plus PR | 83% (79%, 86%) | 83% (80%, 86%) |
| DCV plus PR | 65% (57%, 73%) | 62% (53%, 70%) |
| SOF plus PR | 82% (63%, 100%) | 81% (68%, 95%) |
| SOF plus LDV | 98% (95%, 100%) | - |
| SOF plus SMV | 97% (90%, 100%) | - |
| SOF plus DCV | 100% (95%, 100%) | 96% (88%, 99%) |
| PrOD | - | 89% (79%, 95%) |
| SOF plus LDV with RBV | 97% (95%, 100%) | - |
| SOF plus SMV with RBV | 93% (86%, 100%) | - |
| SOF plus DCV with RBV | 96% (92%, 100%) | 95% (85%, 99%) |
| PrOD with RBV | - | 95% (83%, 99%) |

CI, confidence interval; DCV, daclatasvir; LDV, ledipasvir; PR, pegylated interferon-ribavirin; RBV, ribavirin; SMV, simeprevir; SOF, sofosbuvir; SVR12, sustained virological response at week 12 after the end of treatment; SVR24, sustained virological response at week 24 after the end of treatment
