# Supplementary material for: Efficacy of Second Generation Direct-Acting Antiviral Agents for Treatment Naïve Hepatitis C Genotype 1: A Systematic Review and Network Meta-Analysis
Source: PLoS One. 2015 Dec 31;10(12):e0145953. doi: 10.1371/journal.pone.0145953 (PMC4701000; doi:10.1371/journal.pone.0145953)
Supplement: S4 Table — (PDF) [file pone.0145953.s012.pdf]

**S4 Table. Pooled incidence rate of serious adverse event at entire of treatment**

| Treatment regimens | No. of studies | No. of subjects | No. of having events | Rate (%) of events (95% CI) |
|---|---|---|---|---|
| PR | 9 | 544 | 42 | 7.5 (5.2, 9.7) |
| SMV plus PR | 5 | 1,032 | 55 | 5.0 (3.6, 6.3) |
| DCV plus PR | 2 | 353 | 28 | 7.9 (5.1, 10.7) |
| SOF plus PR | 2 | 144 | 6 | 4.10 (0.0, 9.6) |
| SOF plus LDV | 3 | 903 | 29 | 3.0 (1.3, 4.7) |
| SOF plus DCV | 1 | 100 | 7 | 7.0 (2.0, 12.0) |
| PrOD | 1 | 79 | 2 | 2.5 (0.3, 8.8) |
| SOF plus LDV with RBV | 3 | 669 | 16 | 1.9 (0, 4.5) |
| SOF plus DCV with RBV | 1 | 70 | 2 | 2.9 (1.0, 6.8) |
| PrOD with RBV | 1 | 36 | 1 | 2.8 (0.1, 14.5) |

CI, confidence interval; DCV, daclatasvir; LDV, ledipasvir; PR, pegylated interferon-ribavirin; RBV, ribavirin; SMV, simeprevir; SOF, sofosbuvir
