# Supplementary material for: Efficacy of Second Generation Direct-Acting Antiviral Agents for Treatment Naïve Hepatitis C Genotype 1: A Systematic Review and Network Meta-Analysis
Source: PLoS One. 2015 Dec 31;10(12):e0145953. doi: 10.1371/journal.pone.0145953 (PMC4701000; doi:10.1371/journal.pone.0145953)
Supplement: S5 Table — (PDF) [file pone.0145953.s013.pdf]

S5 Table. Pooled incidence rate of anemia at entire of treatment

Treatment comparison	No. of studies	No. of subjects	No. of having events	Pooled incidence rate (%) (95% CI)
PR	9	544	107	25.4 (14.8, 36.0)
SMV plus PR	5	1,032	263	29.1 (18.6, 39.6)
DCV plus PR	2	353	33	21.5 (0.0, 53.6)
SOF plus PR	2	144	26	17.7 (11.5, 23.9)
SOF plus LDV	3	901	4	0.9 (0.0, 1.8)
PrOD	1	79	1	1.3 (0.0, 6.9)
SOF plus LDV with RBV	3	671	66	9.6 (7.4, 11.9)
PrOD with RBV	1	40	4	10.0 (2.8, 23.7)

CI, confidence interval; DCV, daclatasvir; LDV, ledipasvir; PR, pegylated interferon-ribavirin; RBV, ribavirin; SMV, simeprevir; SOF, sofosbuvir
