# Supplementary material for: Efficacy of Second Generation Direct-Acting Antiviral Agents for Treatment Naïve Hepatitis C Genotype 1: A Systematic Review and Network Meta-Analysis
Source: PLoS One. 2015 Dec 31;10(12):e0145953. doi: 10.1371/journal.pone.0145953 (PMC4701000; doi:10.1371/journal.pone.0145953)
Supplement: S6 Table — (PDF) [file pone.0145953.s014.pdf]

S6 Table. Pooled incidence rate of fatigue at entire of treatment

Treatment comparison	No. of studies	No. of subjects	No. of having events	Rate (%) of fatigue (95% CI)
PR	8	482	229	50.0 (42.9, 57.1)
SMV plus PR	4	892	383	44.8 (37.7, 51.9)
DCV plus PR	2	353	193	54.7 (49.5, 59.9)
SOF plus PR	2	144	86	56.7 (34.8, 78.7)
SOF plus LDV	2	862	191	22.2 (19.4, 24.9)
SOF plus DCV	1	100	39	39.0 (29.4, 48.6)
PrOD	1	79	16	20.3 (12.0, 30.8)
SOF plus LDV with RBV	2	650	236	36.3 (32.6, 40)
SOF plus DCV with RBV	1	70	24	34.3 (23.2, 45.4)
PrOD with RBV	1	40	10	25.0 (12.7, 41.2)

CI, confidence interval; DCV, daclatasvir; LDV, ledipasvir; PR, pegylated interferon-ribavirin; RBV, ribavirin; SMV, simeprevir; SOF, sofosbuvir
